# Supplementary material for: MNI: An enhanced multi-task neighborhood interaction model for recommendation on knowledge graph
Source: PLoS One. 2021 Oct 28;16(10):e0258410. doi: 10.1371/journal.pone.0258410 (PMC8553089; doi:10.1371/journal.pone.0258410)
Supplement: Attachment — Submitted filename: author to respond reviewer 2.docx [file pone.0258410.s001.docx]

Response to Reviewer 2 Comments

**Point 1:** Reference format is not consistent. The authors should double check and revise it

**Response 1:** Thank you for your suggestions. We have modify the reference format so that they are consistent.

**Point 2:** The algorithm 1 on page 10 should be explained more in detail. It is fuzzy when just looking at the algorithm.

**Response 2:** We are grateful for your suggestions. We have added some more explanations to Algorithm 1 so that it is clearer. We explain the whole procedure in steps: reconstruction, recommendation module, cross&compress unit, and KGE module.

**Point 3:** The blocks and colors in the figure 2 is not well explained. Are there any different meaning about different shapes and colors? The author needs to be clearer.

**Response 4:** Thank you very much for your comments. We have added some explanations about the colors and shapes in the figure 2 in the first paragraph under Section 3. Also, we modify some explanations about Figure 2 to be specific.

**Point 4:** About the recommendation module, the author use 2-layer GAT. Could you explain GAT as for the reader is hard to follow.

**Response 5:** We agree with the reviewers comments. We added some explanations about how the GAT works right before the phrase “2-layer GAT”.

**Point 5:** Minor typos, e.g., Missing space between citation and text

**Response 5:** We agree with the reviewers comments.We have double checked through the paper and change the space problem.
